# Breastfeeding during the COVID-19 pandemic – a literature review for clinical practice

**DOI:** 10.1186/s13006-020-00319-3

**Published:** 2020-09-14

**Authors:** Welma Lubbe, Elina Botha, Hannakaisa Niela-Vilen, Penny Reimers

**Affiliations:** 1 NuMIQ – Quality in Nursing and Midwifery, North-West University, 11 Hoffman St, Potchefstroom, South Africa; 2 School of Health, Midwifery, Tampere University of Applied Sciences, Tampere, Finland; 3 Department of Nursing Science, University of Turku, Turku, Finland; 4 Department of Paediatrics and Child Health, University of KwaZulu Natal, Durban, South Africa

**Keywords:** COVID-19, Breastfeeding, Coronavirus; mother-infant dyad, Skin-to-skin contact, Breast milk expressing, Mother-infant separation, Neonatal

## Abstract

**Background:**

The COVID-19 pandemic is disrupting normal life globally, every area of life is touched. The pandemic demands quick action and as new information emerges, reliable synthesises and guidelines for care are urgently needed. Breastfeeding protects mother and child; its health benefits are undisputed and based on evidence. To plan and support breastfeeding within the current pandemic, two areas need to be understood: 1) the clinical characteristics of COVID-19 as it applies to breastfeeding and 2) the protective properties of breastfeeding, including the practice of skin-to-skin care. This review aims to summarise how to manage breastfeeding during COVID-19. The summary was used to create guidelines for healthcare professionals and mothers.

**Methods:**

Current publications on breastfeeding during the COVID-19 pandemic were reviewed to inform guidelines for clinical practice.

**Results:**

Current evidence states that the Coronavirus is not transmitted via breastmilk. Breastfeeding benefits outweigh possible risks during the COVID-19 pandemic and may even protect the infant and mother. General infection control measures should be in place and adhered to very strictly.

**Conclusions:**

Breastfeeding should be encouraged, mothers and infant dyads should be cared for together, and skin-to-skin contact ensured throughout the COVID-19 pandemic. If mothers are too ill to breastfeed, they should still be supported to express their milk, and the infant should be fed by a healthy individual. Guidelines, based on this current evidence, were produced and can be distributed to health care facilities where accessible information is needed.

## Background

The COVID-19 pandemic is creating global disruption, causing markets to plummet and generating many questions in every area of life. Since it affects health in multiple ways, including sexual and reproductive health, publishing in all these areas has increased lately. Not all knowledge is based on scientific evidence, resulting in consequences which could be detrimental rather than providing the needed protection. One area, that needs to be based on scientific evidence, is breastfeeding. Although limited clinical research is available, we can build on what we know about breastfeeding and previous similar infection outbreaks to plan and manage the crisis. This review is intended for healthcare professionals involved with breastfeeding populations, both in an acute care and community health setting. It should not replace clinical judgement or specialist consultation, but rather strengthen clinical management, provide up-to-date evidence and optimize health in infants who may or may not have been exposed to COVID-19. This review has been compiled from information that is currently known about COVID-19 and since new information about the disease is becoming rapidly available, suggestions may be subject to change as additional information is shared.

The novel corona virus (SARS-CoV-2) originated in Wuhan, in Central China in December of 2019 and spread rapidly across China [1–3]. Forty-nine percent of patients who presented with pneumonia were exposed to the Huanan Seafood Wholesale Market [2, 3]. On 5 February 2020, the virus also spread to other countries including Japan, Thailand, Singapore, Republic of Korea, the United States of America and Australia [1]. Currently, every continent across the globe has been affected [3] and the World Health Organization (WHO) declared the outbreak of the disease as an international public health emergency [4].

Although most of the infections occur in adults older than 60 years [3], some pregnant women have also been infected, causing concerns for the management of the perinatal period. A few studies have explored the infection of neonates with SARS-CoV-2 and none showed breastfeeding as method of the transmission of the virus [1, 5–7]. Various research studies reporting primary data have been published on SARS-CoV-2 transmission in the perinatal period. Chen et al. published a retrospective review of medical records of nine infected pregnant woman [5], while Rasmussen et al. published their article on COVID-19 and Pregnancy: What obstetricians need to know [8]. Zhu and colleagues published a clinical analysis of 10 neonates born to mothers with 2019-nCoV pneumonia [1], while Zeng et al. performed a retrospective analysis of 33 neonates born from COVID-19 positive mothers [6]. Schwartz published an analysis of 38 pregnant women with COVID-19 and found no evidence supporting intrauterine or transplacental transmission from infected pregnant women to their foetuses [9]. Aligned with these publications, authoritative bodies released statements regarding COVID-19: the incidence, mortality, prevention and treatment thereof.

Within this valuable, but limited scientific information, in addition to an overload of public information, the question remained: How do we manage breastfeeding in the wake of COVID-19? The authors aim to provide a synthesis on what is currently known about COVID-19, with specific reference to breastfeeding in infants born to healthy, exposed or infected mothers. In addition, the authors aim to provide guidelines for the management of breastfeeding at home and within health facilities, including in the high risk and neonatal intensive care units. To plan and support breastfeeding within the current pandemic, two areas need to be understood: 1) the clinical characteristics of COVID-19 as it applies to breastfeeding and 2) the protective properties of breastfeeding, including the practice of skin-to-skin care. Taking these aspects into account it was possible to compile a clinical guide for both healthcare professionals and breastfeeding mothers.

## Method

The current (15 June 2020) publications on COVID-19 with specific reference to breastfeeding were reviewed to provide evidence-based information for healthcare professionals as well as mothers. Official documents available in English were included due to language abilities of the authors. These included published and in-press clinical research articles, as well as interim guides, expert reviews or guidelines/official statement documents from international associations, including the clinical management interim guides released by the World Health Organization in 2020 and the International Confederation of Midwives’ (ICM) Official Statements of Novel Coronavirus (SARS-CoV-2) and Pregnancy, as well as the Academy of Breastfeeding Medicine (ABM). In addition, articles discussing the characteristics of COVID-19 were included with specific interest in vertical transmission potential in the perinatal period. Articles explaining how breastfeeding could protect against the virus were also included.

The following exclusion criteria were used: Webpages which provide the public with questions and answers, media releases and practice advisories were excluded, since it was based on, and mostly included a repetition of official statements and documents and articles. Country-based webpages were read to compare available data, but not included in the review summary, since it was mostly based on the global recommendations of the WHO and if any discrepancies in management were found, the WHO recommendations were regarded as superior to country recommendations.

### Data analysis

The publications have been reviewed for the characteristics of COVID-19 in the perinatal period, the potential of transmission from infected mother to her breastfeeding infant and proposed management of healthy, exposed and infected mother-infant dyads. The findings were synthesised and a clinical guide with rationale for each suggestion provided (see Table 1) and algorithms for both healthcare providers (Additional file 1) and mothers (Additional file 2) were designed.

## Results

Since the Coronavirus is a novel virus, we have little research to work with and must explore the data that is available, as well as build on knowledge and experience of similar past viral outbreaks, including SARS-CoV-1 and Middle Eastern Respiratory Syndrome (MERS). Furthermore, there is considerable knowledge on the properties of breastmilk which can inform researchers and clinicians of the most suitable route of action, within the limited knowledge on the COVID-19 disease. We will first discuss what is known about COVID-19 specifically within the context of breastfeeding [10] and then look at how breastmilk can be utilized as an intervention to protect infants. Laboratory research on breastfeeding and COVID-19 seems to have been initiated, as evident from social media, however no research on this topic is currently available.

### COVID-19: the disease epidemiology and the breastfeeding population

SARS-CoV-2 is a beta coronavirus, which cause the COVID-19 disease [11]. Currently the mode of transport of SARS-CoV-2 suggests person-to-person transmission which occurs when in close contact with an infected person. The virus is transferred via respiratory droplets produced when coughing and sneezing. Droplets can either land on a healthy individual close to a cavity in the facial area or be inhaled into the lungs of persons in close proximity. It is important to note that the airborne transmission over long distances is unlikely.

In a recent study published in The Lancet, it was stated that all the information available on pneumonia caused by the 2019 novel coronavirus disease was based on information from the general population [2]. The conclusion drawn by Qiao was that the SARS-CoV-2 may have similar pathogenesis to the SARS-CoV1 and therefore we can draw on the management of these previous epidemics to inform current practice guidelines [12].

Vertical transmission refers to the passage of a pathogen from mother to infant during the period before and after birth, including via placental blood during pregnancy, via the birth canal during labour, delivery and during postpartum feeding [1]. In their study Zhu et al. found no evidence of such vertical transmission [1]. In addition, in a retrospective review of clinical records of nine pregnant women by Chen, Guo et al., the authors found limited data available to support the transmission potential of SARS-CoV-2 from mother to child via breastmilk. They assessed the evidence of intrauterine vertical transmission by testing for the presence of the virus in amniotic fluid, cord blood, neonatal throat swab samples and breastmilk samples collected and tested from patients after the first lactation [5]. Although some case studies of infants infected with COVID-19 have been reported, it can be noted that these studies did not test for the presence of the virus in amniotic fluid, cord blood, neonatal throat swabs or breastmilk [7, 13–15] and included only three infants in total, indicating further investigation is needed. A few case studies were published from Spain [16], Vietnam [13], China [15] and USA [17], and none of these reported transmissions of the SARS-CoV-2 via breastmilk. A systematic review by Duran et al. and a study by Lu and Shi also reported that breastmilk does not appear to be a method of transmission of the virus [18, 19]. Samples of breastmilk from 18 women infected with SARS-CoV-2 were evaluated and although SARS-CoV-2 RNA was detected in one sample of milk, the follow-up culture of the same sample was negative. It is likely that the SARS-CoV-2 RNA that was found does not contain replication-competent virus and so is unlikely to infect an infant [20]. Findings from this group of cases suggest that there is currently no evidence to show that respiratory viruses can be transmitted through breast milk.

### Breastfeeding and breastmilk properties

Breastfeeding protects neonates, infants and children against morbidity and death [21, 22]. The protective effect is particularly strong against infectious diseases, due to the direct transfer of antibodies as well as anti-infective factors and long-lasting transfer of immunological competence and memory [23].

Breastfeeding has both short and long-term benefits for the mother and her infant. To benefit from the protective factors in breast milk, every effort should be made to support and enable early and immediate initiation of breastfeeding. Not only does the early initiation decrease neonatal deaths, but together with frequent breastfeeding, ensures that the breastfeeding dyad are not separated. Early initiation of breastfeeding significantly increases the breastfeeding rates in healthy term infants at one to 4 month’s age by stimulating hormones and facilitating bonding [24–26]. The Lancet Breastfeeding Series (2016) reported that scaling up breastfeeding could prevent around 823,000 child deaths annually [21]. Breastfeeding reduces 64% of morbidity and mortality in diarrhoea, 74% in the severity of RSV and its hospitalization with 72% [22]. This demonstrates the protective benefits of breastfeeding, which pertain to COVID-19 pandemic.

The neonate has an immature immune system and colostrum, a powerful immune booster, protects infants from infections by means of bioactive factors and secretory IgA antibodies. Breast milk with its abundant source of immunoglobulins, lactoferrin, lysozyme and cytokines play an important role in absorbing and engulfing harmful micro-organisms and targeting specific bacteria and providing protection by regulating the immune response [27]. Human milk oligosaccharides, abundant in human milk, shape the microbiome, provide probiotics and modulate the developing immune system also displaying anti-adhesive effects for bacterial antigens [28–30]. All the above are compelling reasons for every infant to receive only breast milk and preferably their own mothers’ milk.

The best way to promote successful breastfeeding, is to ensure that the mother-infant dyad is kept together, and skin-to-skin contact is supported and encouraged [31]. Skin-to-skin is the safest and best transition for mothers and their infants to a new life together. Ensuring it happens immediately after birth, the infant’s microbiome can develop from the mother’s flora, so beneficial during a pandemic [32]. Skin-to-skin also increases blood glucose levels 75–90 min after birth, improves cardiorespiratory stability [26] and significantly reduces stress levels in the infant and mother [33]. Keeping mother and infant together can reduce birth stress and even prevent neurodevelopmental disorders in the infant. The smell, touch and voice of the mother naturally calms the infant [34].

### Suggested practice guidelines

It should be noted that the WHO interim guidance documents on the management of COVID-19 has been informed by evidence-based guidelines also published by the WHO, such as the *Infection prevention and control of epidemic- and pandemic-prone acute respiratory diseases in health care* [35], as well as current information on COVID-19. General infection prevention measures should therefore always be taken, in all circumstances, with special attention to droplet protection. Guidance about the need for exposed and infected breastfeeding mothers to practice hand and respiratory hygiene has been given by many organisations internationally and experts in the field [36–39]. Additional suggestions are provided based on the current knowledge of COVID-19. Based on this information, and her right to choose, the mother can make an informed choice regarding breastfeeding during the pandemic [40]. Measures for expressing breastmilk should also be applied as in normal situations, however, no equipment should be shared between mothers. All equipment used to express milk, should be rinsed with cold water and secondly washed with warm water and soap and thirdly, sterilized. In case of an epidemic it may be advisable to sterilise equipment after each use, instead of once in 24 h [27]. Another additional measure is that if a mother is potentially exposed or tested positive for COVID-19, she should use a dedicated breast pump and not share one with other mothers. She should also express in the area in which she has been isolated. A practical suggestion may be that the mother express in her container in isolation and then decant the expressed milk into a clean container held by a healthy person with appropriate protective clothing, including masks and gloves, to prevent the virus from spreading via the surface of the container. In this manner a clean container can then also be stored/milk frozen for later use without the risk of contaminating other containers with milk [41]. There has been much debate on ways to reduce the risk of the external contamination of expressed human milk containers. Marinelli and Lawrence, advise bottles to be wiped down with a viricidal agent or a dilution of 1:10 diluted bleach (sodium hypochlorite [NaOCl]) and bottles stored in separate bins for each infant in refrigerators [37]. However, concerns were expressed about the necessity to do this as no proof of contamination of bottle surfaces exists [42].

Table 1 provide a synthesis of the evidence supported recommendations for breastfeeding amidst COVID-19, based on the latest evidence as available on 15 June 2020. Information about SARS-CoV-2 transmission is emerging daily, and the latest information should always be considered in clinical decision making.

**Table 1:** Suggested guidelines for breastfeeding during COVID-19 pandemic with supporting evidence

Category	Suggested guideline	Rationale/remarks
All infants	Avoiding separation of mother-infant dyads, including infection control precautions.	Separation of a mother and her infant may have potential detrimental effects on feeding and bonding [18, 38, 40, 43–45].
Infant from healthy or non-symptomatic mother	Initiate and continue breastfeeding.	Breastfeeding is the optimal source of nutrition and protection for infants [46, 47].
Infant of mother with suspected COVID-19 (potentially exposed and/or symptomatic, but well enough to breastfeed)	Continue breastfeeding while applying infection control protocols with specific attention to droplet protection.	Mechanisms of breastmilk protect the infant [40, 43–45]. Respiratory viruses are not transmitted in breastmilk [1, 5, 38]. Direct transfer of antibodies into breastmilk [43]. Anti-infective factors transfer to breastmilk [43]. Immunological competence and memory in breastmilk [43].
	Continue breastfeeding.	The virus is not transmitted via breastmilk [1, 5, 15, 38, 43, 44].
	All laboratory confirmed cases are isolated and cared for in a health care facility. Isolate mother-infant dyad together.	Avoid person-to-person transmission of the virus [35, 40].
	Wear a mask during feeding.	To avoid coughing or sneezing on the baby while feeding at the breast [38, 43, 44, 47, 48].
Infant of mother who test positive for COVID-19 (mother well enough to breastfeed)	Continue breastfeeding.	No virus detected in amniotic fluid [5, 15]. No virus detected in cord blood [5, 15]. No virus detected in neonatal throat swabs [5, 15]. No virus detected in breastmilk after first breastfeeding [5, 15].
Positive mother with mild symptoms	Isolate mother-infant dyad at home.	Avoid person-to-person transmission of the virus [35].
	Wear a mask during feeding.	To avoid coughing or sneezing on the infant while feeding at the breast [38, 43, 44, 47, 48].
Positive mother with severe symptoms (can breastfeed)	Hospitalize mother-infant dyad.	All laboratory confirmed cases are isolated and cared for in a health care facility [35, 40].
	Isolate mother-infant dyad together in hospital.	Avoid person-to-person transmission of the virus [35, 40].
	Conduct a risk/benefits discussion between neonatologists and families to individualise infant care in the case of infants requiring neonatal care.	Admission to the neonatal unit may be required in infants that are born to COVID-19 positive mothers and/or have symptoms. This may require close observation and may lead to separation, however, not necessarily to discontinuation of breastfeeding [38].
		Temporary separation may be needed in case of mothers who need medical care in hospital -continue to express milk [40, 47].
Infant of mother who test positive for COVID-19 (mother too ill to breastfeed)	Express milk and feed with clean cup or spoon.	Prevent accidental transmission of pathogens via surface areas [35, 44].
	*Practical note: Mother can decant milk from her container into a clean container held by a healthy person to prevent transmission via the containers surface.	
	Wear a mask during expressing.	Prevent accidental transmission of pathogens via air [35, 44, 47].
	Ask a healthy person to feed the baby expressed milk.	Prevent accidental transmission of pathogens via person-to-person contact or air [38, 47].
	In case of separation, ensure appropriately trained mental health and psychosocial support for parents.	The prevalence of common mental disorders in the antenatal and postpartum period is high [43].
**Expressing breastmilk:**	*Assumption*: COVID-19 is not found in breastmilk, therefore general expressing guidelines apply.	
	Apply all general infection prevention measures.	Prevent accidental transmission of pathogens [35, 38, 47]
	PUI and positive mothers should express in the area where they are isolated.	Prevent potential infection of non-infected areas and persons.
	Do not share equipment for breastmilk expressing.	Milk particles can be left within any parts of the equipment and transferred to the next person. Any pathogens surviving on surfaces can be transferred via equipment [38, 49].
	Rinse all expressing equipment in clean, cold running water before sterilising.	Prevent proteins in milk to coagulate due to heat, cold water removes milk residue [38].
	Decant expressed milk into a clean container held by a healthy person wearing protective clothing.	Prevent transmission of the virus via container surface area potentially infected by positive mother via surfaces [42}.
	Wipe down bottles with a viricidal agent of 1:10 diluted bleach.	Kill pathogens on surfaces, such as bottles handles by infected person [37].
	Wash expressing equipment with hot soapy water with domestic detergent, rinse under running water and dry with a paper towel.	Kill pathogens [38, 47].
	Sterilise equipment after each use [42].	Kill pathogens [38].
**General infection control measures**		
Personal hygiene	Washing hands often for 20 s with soap and water or using alcohol-based hand rub (based on 80% ethanol or 75% 2-propanol).	Prevent accidental transmission of pathogens via person-to-person contact [4, 47, 50].
Personal hygiene	Cover mouth and nose with a disposable tissue or elbow when coughing and sneezing.	Prevent accidental transmission of pathogens via air [4, 14].
Personal hygiene	Wash hands before and after contact with the infant.	Prevent accidental transmission of pathogens via person-to-person contact [38, 43, 44, 47, 48].
Environment	Thoroughly cook meat and eggs.	Prevent accidental transmission of pathogens in food sources [4].
Environment	Clean/disinfect contaminated surfaces immediately.	Prevent accidental transmission of pathogens via surfaces [43, 44, 49].
	Surface disinfection with 0.1% sodium hypochlorite or 71% ethanol reduces coronavirus infectivity on surfaces within 1-min exposure time.	Human coronaviruses can remain infectious on surfaces for up to 9 days [49].
Social distance	Keep mother and baby together and practice safe skin-to-skin contact and rooming-in.	Immediately after birth, during establishment of breastfeeding and whether mother or infant have suspected, probable, or confirmed COVID-19, 24 h/day [18, 43, 51].
Social distance	Avoid close contact with anyone who is coughing and/or sneezing.	Prevent accidental transmission of pathogens via air [4].
Social distance	Avoid crowds of people such as church gatherings, shopping malls and large events.	Prevent accidental transmission of pathogens via person-to-person contact or air [4].
Social distance	Do not allow anyone to kiss the infant and ask them to disinfect their hands when they come into the house or plan to touch the infant.	Prevent accidental transmission of pathogens via person-to-person contact [4].
Social distance	Do not allow anyone who is ill to visit the mother and infant.	Prevent accidental transmission of pathogens via person-to-person contact or air [4].
**Mental health of mothers during COVID-19 pandemic**	No need for alarm.	Children (aged 1–16 years) accounted for roughly 5% of total patients with COVID-19 in China, but frequently do not have a notable disease and no transfer via breastmilk has been confirmed [1, 5, 7, 13, 52].
	Use self-isolation time to bond with infant at home.	Bonding improves relationships and mental health and overall wellbeing [51].

Based on the evidence presented in Table 1, a visual presentation for quick clinical reference is presented as an algorithm for healthcare professionals (Additional file 1) and mothers (Additional file 2) to support in decision-making regarding breastfeeding practices in the wake of COVID-19.

## Discussion

Global measures to control the spread of the coronavirus should be applied in everyday situations to prevent and stop the spread of pathogens. These measures include personal hygiene and social distancing, which should be applied to all infants, to prevent them from contracting the illness (refer to Table 1). This review aimed to map the current evidence-based literature about breastfeeding and COVID-19.

Continued breastfeeding and zero-separation of the mother-infant dyad appears to be the best practice in this situation. Considering the current evidence, it is not common for respiratory viruses to be transmitted via breast milk and transmission such as this has not been demonstrated in infants who contracted COVID-19 or were born to mothers who tested positive for the virus [16, 53]. In addition, the properties in breast milk can protect the infant [10, 43].

In their Practice Advisory on the Novel Coronavirus 2019 (SARS-CoV-2), the American College of Obstetricians and Gynecologists (ACOG), state that infants born to mothers with confirmed COVID-19, should be considered persons under investigation (PUI), and as such should be isolated according to the Infection Prevention and Control Guidance for PUIs [54, 55]. They further state that confirmed or PUI mothers should be separated from their infants, in separate rooms until the maternal transmission-based precautions are discontinued. The National Health Commission of China in their notice: strengthening maternal disease treatment and safe midwifery during the prevention and control of new coronavirus pneumonia, in February 2020 [56], also followed this recommendation of separation for at least 14 days and not breastfeeding. However, in the next section of the ACOG advisory on breastfeeding it is stated that breastfeeding should be initiated and continued, since currently it seems that this respiratory virus cannot be transmitted through breastmilk [54]. American Academy of Pediatrics (AAP), 2020 and the Centers for Disease Control and Prevention (CDC), also suggest that infected mothers and their infants be temporarily separated and once home should maintain a distance of at least 6 feet (2 m) between them, with mothers expressing to provide breastmilk for their infants [57, 58].

The ICM [4] concur with UNICEF, the ABM and RCOG in that currently there is no evidence that respiratory viruses can be transmitted via breast milk. Therefore, breastfeeding should be continued while general infection control measures are applied in symptomatic mothers and when mothers are too ill to breastfeed. Mothers who are too ill to breastfeed could express milk, and a healthy individual could then cup, or spoon feed the infant.

The authors agree that breastfeeding should be continued, since the protective benefits of breastmilk far outweigh the risk of potential pathogen transmission. In addition, we want to highlight that mother and infant separation has negative effects on the mental and physical health of both mothers and infants [34, 59] and should therefore be limited to extreme situations and supported by good evidence or clinical reasoning (see Table 1).

## Conclusions

Breastfeeding is the best protective measure available for healthy and at-risk infants and their mothers during the COVID-19 pandemic. Therefore, breastfeeding should not be interrupted, mothers and infants should not be separated, and skin-to-skin contact should not be discontinued. While ensuring normality as far as possible, general infection control measures should be in place and adhered to very strictly. In exposed or infected mother’s additional droplet protection should be taken by mother by wearing a (see-through), surgical face mask when handling and feeding her infant. When mothers are too ill to breastfeed, they should still be supported to express their milk, and the infant should be fed by a healthy individual. Breastfed infants have an advantage receiving additional protection against SARS-CoV-2. Based on the current evidence, it seems that the virus is not transmitted via breastmilk. For this reason, the benefits of breastmilk outweigh the risk of breastfeeding cessation and of a potential transmission of the coronavirus [38].

## Supplementary information

**Additional file 1.** Professional guide to breastfeeding and COVID-19.

**Additional file 2.** Mothers guide to breastfeeding and COVID-19.

## Data Availability

Not applicable

## Abbreviations

SARS-CoV-2: Severe acute respiratory syndrome coronavirus 2
MERS: Middle Eastern Respiratory Syndrome
COVID-19: Coronavirus disease 2019
2019 nCoV: 2019 Novel Coronavirus
RSV: Respiratory Syncytial Virus
WHO: World Health Organization

## References

[1] Zhu H, Wang L, Fang C, Peng S, Zhang L, Chang G (2020). Clinical analysis of 10 neonates born to mothers with 2019-nCoV pneumonia. Transl Pediatr.

[2] Chen N, Zhou M, Dong X, Qu J, Gong F, Han Y (2020). Epidemiological and clinical characteristics of 99 cases of 2019 novel coronavirus pneumonia in Wuhan, China: a descriptive study. Lancet.

[3] Li Q, Guan X, Wu P, Wang X, Zhou L, Tong Y (2020). Early transmission dynamics in Wuhan, China, of novel coronavirus-infected pneumonia. N Engl J Med.

[4] International Confederation of Midwives, (ICM). Official statements on novel coronavirus (COVID-19) and pregnancy. Available at: https://www.internationalmidwives.org/icm-news/unfpa-statement-on-novel-coronavirus-(covid-19)-and-pregnancy.html. Issued 7 Mar 2020. Accessed 20 Mar 2020.

[5] Chen H, Guo J, Wang C, Luo F, Yu X, Zhang W (2020). Clinical characteristics and intrauterine vertical transmission potential of COVID-19 infection in nine pregnant women: a retrospective review of medical records. Lancet.

[6] Zeng L, Xia S, Yuan W, Yan K, Xiao F, Shao J (2020). Neonatal early-onset infection with SARS-CoV-2 in 33 neonates born to mothers with COVID-19 in Wuhan, China. JAMA Pediatr.

[7] Dong L, Tian J, He S, Zhu C, Wang J, Liu C (2020). Possible vertical transmission of SARS-CoV-2 from an infected mother to her newborn. JAMA..

[8] Rasmussen SA, Smulian JC, Lednicky JA, Wen TS, Jamieson DJ (2020). Coronavirus disease 2019 (COVID-19) and pregnancy: what obstetricians need to know. Am J Obstet Gynecol.

[9] Schwartz DA (2020). An analysis of 38 pregnant women with COVID-19, their newborn infants, and maternal-fetal transmission of SARS-CoV-2: maternal coronavirus infections and pregnancy outcomes. Arch Pathol Lab Med.

[10] Centers for Disease Control and Prevention, (CDC). Interim infection prevention and control recommendations for patients with suspected or confirmed coronavirus disease 2019 (COVID-19) in healthcare settings. Available at: https://www.cdc.gov/coronavirus/2019-ncov/infection-control/control-recommendations.html. Updated 18 May 2020. Accessed 15 June 2020.

[11] Hong H, Wang Y, Chung HT, Chen CJ (2020). Clinical characteristics of novel coronavirus disease 2019 (COVID-19) in newborns, infants and children. Pediatr Neonatol.

[12] Qiao J (2020). What are the risks of COVID-19 infection in pregnant women?. Lancet.

[13] Le TH, Nguyen VL, Tran MD, Do TH, Tran TH, Le TY (2020). The first infant case of COVID-19 acquired from a secondary transmission in Vietnam. Lancet Child Adolesc Health.

[14] Zeng H, Xu C, Fan J, Tang Y, Deng Q, Zhang W (2020). Antibodies in infants born to mothers with COVID-19 pneumonia. JAMA..

[15] Fan C, Lei D, Fang C, Li C, Wang M, Liu Y, et al. Perinatal transmission of COVID-19 associated SARS-CoV-2: should we worry? Clin Infect Dis. 2020. 10.1093/cid/ciaa226.

[16] Díaz AC, Maestro LM, Pumarega MT, Antón FB, Alonso PCR (2020). First case of neonatal infection due to SARS-CoV-2 in Spain. An Pediatr.

[17] Dumpa V, Kamity R, Vinci AN, Noyola E, Noor A (2020). Neonatal coronavirus 2019 (COVID-19) infection: a case report and review of literature. Cureus..

[18] Duran P, Berman S, Niermeyer S, Jaenisch T, Forster T, Gomez Ponce de Leon R (2020). COVID-19 and newborn health: systematic review. Rev Panam Salud Publica.

[19] Lu Q, Shi Y (2020). Coronavirus disease (COVID-19) and neonate: what neonatologist need to know. J Med Virol.

[20] Chambers CD, Krogstad P, Bertrand K, Contreras D, Bode L, Tobin N, et al. Evaluation of SARS-CoV-2 in breastmilk from 18 infected women. medRxiv. 2020. 10.1101/2020.06.12.20127944. 10.1001/jama.2020.15580 PMC7439212 32822495

[21] Victora CG, Bahl R, Barros AJD, França GVA, Horton S, Krasevec J (2016). Breastfeeding in the 21st century: epidemiology, mechanisms, and lifelong effect. Lancet.

[22] American Academy of Pediatrics, (AAP) (2012). Breastfeeding and the use of human milk. Pediatrics.

[23] Hanson LA (1998). Breastfeeding provides passive and likely long-lasting active immunity [published correction appears in Annals of Allergy, Asthma & Immunology. 1999 May;82(5):478]. Ann Allergy Asthma Immunol.

[24] Khan J, Vesel L, Bahl R, Martines J (2015). Timing of breastfeeding initiation and exclusivity of breastfeeding during the first month of life: effects on neonatal mortality and morbidity—a systematic review and meta-analysis. Matern Child Health J.

[25] Jaafar SH, Ho JJ, Lee KS (2016). Rooming-in for new mother and infant versus separate care for increasing the duration of breastfeeding. Cochrane Database Syst Rev.

[26] Moore ER, Anderson GC, Bergman N, Dowswell T (2016). Early skin-to-skin contact for mothers and their healthy newborn infants. Cochrane Database Syst Rev.

[27] Hamosh M (2001). Bioactive factors in human milk. Pediatr Clin N Am.

[28] Coppa VG, Gabrielli VO, Zampini VL, Galeazzi VT, Ficcadenti VA, Padella VL (2011). Oligosaccharides in 4 different milk groups, Bifidobacteria, and Ruminococcus Obeum. J Pediatr Gastroenterol Nutr.

[29] Morrow AL, Ruiz-Palacios G, Altaye M, Jiang X, Lourdes Guerrero M, Meinzen-Derr J (2004). Human milk oligosaccharides are associated with protection against diarrhea in breast-fed infants. J Pediatr.

[30] Williams JE, Price WJ, Shafii B, Yahvah KM, Bode L, McGuire MA (2017). Relationships among microbial communities, maternal cells, oligosaccharides, and macronutrients in human Milk. J Hum Lact.

[31] Karimi FZ, Sadeghi R, Maleki-Saghooni N, Khadivzadeh T (2019). The effect of mother-infant skin to skin contact on success and duration of first breastfeeding: a systematic review and meta-analysis. Taiwan J Obstet Gynecol.

[32] Dumas L (2015). Safe skin-to-skin contact between mother and baby. Procedure and important notes.

[33] Mörelius E, Theodorsson E, Nelson N (2005). Salivary cortisol and mood and pain profiles during skin-to-skin care for an unselected group of mothers and infants in neonatal intensive care. Pediatrics..

[34] Császár-Nagy N, Bókkon I (2018). Mother-newborn separation at birth in hospitals: a possible risk for neurodevelopmental disorders?. Neurosci Biobehav Rev.

[35] World Health Organization, (WHO). Infection prevention and control of epidemic- and pandemic-prone acute respiratory infections in health care. Available at: https://apps.who.int/iris/handle/10665/112656. Accessed 20 Mar 2020. 24983124

[36] Centers for Disease Control and Prevention. Coronavirus Disease 2019 (COVID 19). Pregnancy & Breastfeeding. Information about coronavirus disease 2019. Available at: https://www.cdc.gov/coronavirus/2019-ncov/prepare/pregnancy-breastfeeding.html. Updated June 2020. Accessed 17 Jun 2020.

[37] Marinelli KA, Lawrence RW. Safe handling of containers of expressed human milk in all settings during the SARS-CoV-2 (COVID-19) pandemic. J Hum Lact. 2020. 10.1177/0890334420919083. 10.1177/0890334420919083 PMC7411511 32242762

[38] Royal College of Obstetricians and Gynaecologists, Royal College of Midwives, Royal College of Paediatrics and Child Health, Public Health England and Health Protection Scotland (2020). Coronavirus (COVID-19) infection in pregnancy. Information for healthcare professionals Version 10.1.

[39] World Health Organization, (WHO). Breastfeeding and COVID-19 for health care workers. Available at: https://www.who.int/docs/default-source/maternal-health/faqs-breastfeeding-and-covid-19.pdf?sfvrsn=d839e6c0_5. Issued 12 May 2020. Accessed June 2020.

[40] Academy of Breastfeeding Medicine, (ABM). ABM Statement on coronavirus 2019 (COVID-19). Available at: https://www.bfmed.org/abm-statement-coronavirus. Issued 10 Mar 2020. Accessed 15 Mar 2020.

[41] Moro GE, Bertino E. Breastfeeding, human milk collection and containers, and human milk banking: hot topics during the COVID-19 pandemic. J Hum Lact. 2020. 10.1177/0890334420934391. 10.1177/0890334420934391 32510263

[42] Mitchell KB, Weinstein SR. Concerns regarding the article entitled safe handling of containers of expressed human milk in all settings during the SARS-CoV-2. J Hum Lact. 2020. 10.1177/0890334420922580. 10.1177/0890334420922580 32324442

[43] World Health Organization (WHO) (2020). Clinical management of severe acute respiratory infection when novel coronavirus (nCoV) infection is suspected.

[44] UNICEF. Coronavirus disease (COVID-19): What parents should know how to protect yourself and your children. Available at: https://www.unicef.org/stories/novel-coronavirus-outbreak-what-parents-should-know. Issued 29 Mar 2020. Accessed 20 June 2020.

[45] Royal College of Obstetricians and Gynaecologists (2020). Coronavirus (COVID-19) infection and pregnancy. Information for pregnant women and their families.

[46] World Health Organization, (WHO). Infant and young child feeding. Available at: https://www.who.int/news-room/fact-sheets/detail/infant-and-young-child-feeding. Updated 1 Apr 2020. Accessed 17 July 2020.

[47] Carvalho WB, Gibelli MABC, Krebs VLJ, Calil VMLT, Johnston C (2020). Expert recommendations for the care of newborns of mothers with COVID-19. Clinics..

[48] Wei M, Yuan J, Liu Y, Fu T, Yu X, Zhang Z (2020). Novel coronavirus infection in hospitalized infants under 1 year of age in China. JAMA..

[49] Kampf G (2020). Potential role of inanimate surfaces for the spread of coronaviruses and their inactivation with disinfectant agents. Infect Prev Pract.

[50] Siddharta A, Pfaender S, Vielle NJ, Dijkman R, Friesland M, Becker B (2017). Virucidal activity of World Health Organization–recommended formulations against enveloped viruses, including Zika, Ebola, and emerging coronaviruses. J Infect Dis.

[51] Widstrom A-M, Brimdyr K, Svensson K, Cadwell K, Nissen E (2019). Skin-to-skin contact the first hour after birth, underlying implications and clinical practice. Acta Paediatr.

[52] Kelvin AA, Halperin S (2020). COVID-19 in children: the link in the transmission chain. Lancet Infect Dis.

[53] Aghdam K, Jafari N, Eftekhari K (2020). Novel coronavirus in a 15-day-old neonate with clinical signs of sepsis, a case report. Infect Dis.

[54] The American College of Obstetricians and Gynecologists, (ACOG). Novel coronavirus 2019 (COVID-19). Available at: https://www.acog.org/clinical/clinical-guidance/practice-advisory/articles/2020/03/novel-coronavirus-2019. Updated 1 July 2020. Accessed 18 July 2020.

[55] Centers for Disease Control and Prevention, (CDC). CDC infection prevention and control guidance for PUIs. Available at: https://www.cdc.gov/coronavirus/2019-ncov/php/guidance-evaluating-pui.html. Issued 10 Apr 2020. Accessed 18 July 2020.

[56] National Health Commission of the People’s Republic of China. National Health Commission (NHC) of the People’s Republic of China. Notice on strengthening maternal disease treatment and safe midwifery during the prevention and control of new coronavirus pneumonia. Available at: http://www.nhc.gov.cn/xcs/zhengcwj/202002/4f80657b346e4d6ba76e2cfc3888c630.shtml. Accessed 8 Feb 2020.

[57] American Academy of Pediatrics, (AAP). Guidance on breastfeeding during the COVID-19 pandemic. Available at: https://www.aappublications.org/news/2020/04/23/covid19breastfeeding042320. Issued 23 Apr 2020. Accessed 19 June 2020.

[58] Centers for Disease Control and Prevention, (CDC). Evaluation and management considerations for neonates at risk for COVID-19. Available at: https://www.cdc.gov/coronavirus/2019-ncov/hcp/caring-for-newborns.html. Updated 20 May 2020. Accessed 19 June 2020.

[59] Bystrova K, Ivanova V, Edhborg M, Matthiesen AS, Ransjö-Arvidson AB, Mukhamedrakhimov R (2009). Early contact versus separation: effects on mother-infant interaction one year later. Birth..

