# Supplementary material for: Breastfeeding during the COVID-19 pandemic – a literature review for clinical practice
Source: Int Breastfeed J. 2020 Sep 14;15:82. doi: 10.1186/s13006-020-00319-3 (PMC7487446; doi:10.1186/s13006-020-00319-3)
Supplement: Supplementary file 1 — Additional file 1. Professional guide to breastfeeding and COVID-19. [file 13006_2020_319_MOESM1_ESM.pdf]

BREASTFEEDING AND COVID-19

A guide for professionals

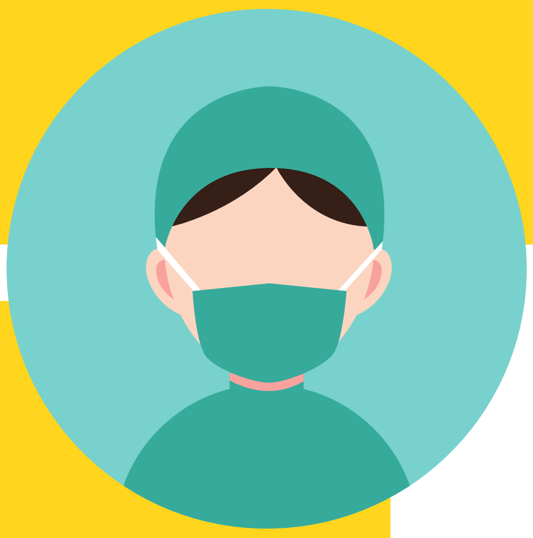

?

DOES THE MOTHER HAVE SYMPTOMS?

Fever or one or more of the following: Cough, difficult breathing or shortness of breath or gastrointestinal symptoms.

Yes ↓

No → **Encourage to breastfeed, and protect herself!** ↓

- **Wash hands with soap frequently, for at least 20 seconds.**
- **Avoid touching own or baby's face.**
- **Cover mouth and nose with a tissue or elbow when coughing and sneezing.**
- **Don't allow anyone who is ill to visit mother and infant.**
- **Avoid crowds, consider self-isolation.**
- **Get information from trusted sources only.**

1 ASSESS THE MOTHER AND INFANT

Take infection control precautions.

2 ENCOURAGE BREASTFEEDING -DON'T SEPARATE INFANT AND MOTHER!

COVID-19 is not transmitted to the infant via breast milk. Make sure a healthy adult cares for the infant's other needs. Remind mother of hygiene measures, such as washing hands with soap before and after feeding and wearing a mask while breastfeeding. If necessary, isolate mother-infant dyad together.

3 IF MOTHER IS UNABLE TO BREASTFEED

Enable mother to express breast milk 8-12 times/24 hours, to maintain milk production. Have a healthy adult feed and care for the infant. Apply all general infection prevention measures. Wash and sterilise equipment after each use and do not share equipment between mothers. If mother-infant dyad is separated, ensure mental health and psychosocial support.

4 ENSURE SAFE SKIN-TO-SKIN CONTACT AND ROOMING IN

This helps to maintain a bond between mother and infant, and can keep up milk production. Bonding improves relationships, mental health and overall well-being. Encourage safe interaction, such as eye contact and chats even when mother is wearing a mask.

Please note:
Information about COVID-19 transmission is emerging daily. These guidelines are based on latest evidence during publication time.

EVIDENCE SUMMARY BY:

Lubbe W, Botha E, Niela-Vilen H & Reimers P.
Breastfeeding during the COVID-19 pandemic – a literature review for clinical practice. 2020.
DOI : 10.1186/s13006-020-00319-3
