# Supplementary material for: Breastfeeding during the COVID-19 pandemic – a literature review for clinical practice
Source: Int Breastfeed J. 2020 Sep 14;15:82. doi: 10.1186/s13006-020-00319-3 (PMC7487446; doi:10.1186/s13006-020-00319-3)
Supplement: Supplementary file 2 — Additional file 2. Mothers guide to breastfeeding and COVID-19. [file 13006_2020_319_MOESM2_ESM.pdf]

BREASTFEEDING AND COVID-19

A guide for mothers

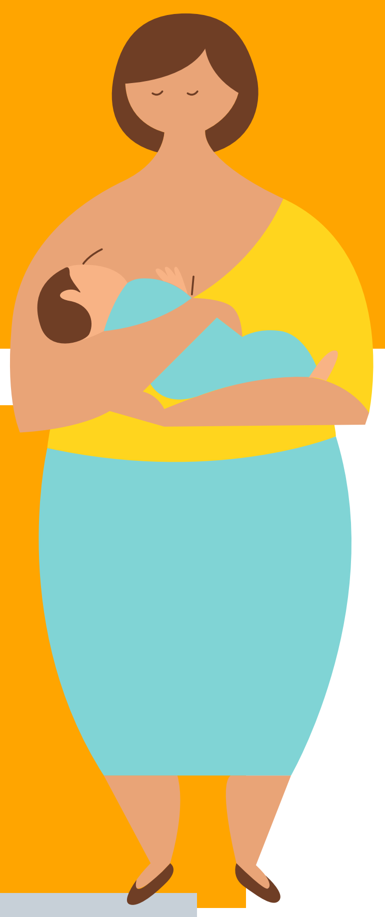

DO YOU HAVE SYMPTOMS?

Fever or one or more of the following: Cough, difficulty breathing or shortness of breath or gastrointestinal symptoms.

Yes

No → **Keep on breastfeeding, protect yourself!**

- **Wash your hands with soap frequently, for at least 20 seconds.**
- **Avoid touching your or baby's face.**
- **Cover your mouth and nose with a tissue or elbow when coughing and sneezing.**
- **Don't allow anyone who is ill to visit you and your baby.**
- **Avoid crowds, consider self-isolation.**
- **Get information from trusted sources only.**

1 CONTACT YOUR HEALTH PROVIDER

Seek medical care by calling them first.

2 CONTINUE BREASTFEEDING -DON'T SEPARATE FROM BABY, EVEN IF YOU HAVE BEEN TESTED POSITIVE

COVID-19 is not transmitted to your baby via breast milk. Ask help from a healthy adult to care for the baby's other needs. Wash hands with soap before and after feeding, wear a mask while breastfeeding, stay isolated together and avoid other people. Don't kiss your baby.

3 IF EXPRESSING MILK... (IF YOU ARE TOO ILL OR SEPARATED FROM YOUR BABY)

Expressing milk can keep up your milk production if you are unable to breastfeed. Use only clean equipment. Never share equipment with other mothers. After use, rinse all equipment in clean, cold, running water and sterilise after each use. Wash the pump with warm water and soap (follow pump manufacturer's directions).

4 CONTINUE SAFE SKIN-TO-SKIN CONTACT AND ROOMING-IN

This helps to keep a bond with your baby and to keep up milk production. Your baby will enjoy eye contact, chats and smiles even when you wear a mask.

Please note:
Information about COVID-19 transmission is emerging daily. These guidelines are based on latest evidence during publication time.

EVIDENCE SUMMARY BY:

Lubbe W, Botha E, Niela-Vilen H & Reimers P.
Breastfeeding during the COVID-19 pandemic – a literature review for clinical practice. 2020.
DOI : 10.1186/s13006-020-00319-3
